# Food Microbiology

**DOI:** 10.1155/2019/8039138

**Published:** 2019-04-04

**Authors:** Marta Laranjo, María de Guía Córdoba, Teresa Semedo-Lemsaddek, Maria Eduarda Potes

**Affiliations:** ^1^Instituto de Ciências Agrárias e Ambientais Mediterrânicas (ICAAM), Universidade de Évora, Pólo da Mitra, Ap. 94, 7006-554 Évora, Portugal; ^2^Universitary Institute for Research in Agroalimentary Resources (INURA), Escuela de Ingenierías Agrarias, University of Extremadura, Avda. Adolfo Suárez s/n, 06007 Badajoz, Spain; ^3^CIISA – Centre for Interdisciplinary Research in Animal Health, Faculty of Veterinary Medicine, University of Lisbon, Pólo Universitário da Ajuda, 1300-477 Lisbon, Portugal; ^4^Departamento de Medicina Veterinária, Escola de Ciências e Tecnologia, Universidade de Évora, Pólo da Mitra, Ap. 94, 7006-554 Évora, Portugal

Food microbiology comprehends the study of microorganisms that colonise, modify, and process, or contaminate and spoil food. It is one of the most diverse research areas within microbiology. It comprises a wide variety of microorganisms including spoilage, probiotic, fermentative, and pathogenic bacteria, moulds, yeasts, viruses, prions, and parasites. It deals with foods and beverages of diverse composition, combining a broad spectrum of environmental factors, which may influence microbial survival and growth.

Food microbiology includes microorganisms that have beneficial or deleterious effects on food quality and safety and may therefore be of concern to public health.

Among the 39 submitted manuscripts, 22 have been selected to be part of this special issue on food microbiology in a broad sense.

This special issue includes studies dealing with antibioresistance in bacteria isolated from food products. M. Kang and B. Wei studied the molecular basis of macrolide resistance in* Campylobacter* strains isolated from poultry in South Korea, highlighting the importance of elucidating the mechanisms underlying resistance development during chicken growth to prevent and control macrolide resistance in* Campylobacter*. G. Liu* et al.* characterised* Escherichia coli* isolates from bovine mastitis exposed to cephalothin or ceftazidime and concluded that the exposure to cephalosporins at sub-MIC levels induced resistant* E. coli* and therefore recommend their careful use in the treatment of clinical* E. coli* mastitis. G. Caruso and coworkers characterised the extraintestinal fluoroquinolone-resistant* E. coli* populations isolated from poultry, beef, and pork meat, revealing a potential zoonotic risk, because meat is a source of resistant bacterial strains. M. Pate and colleagues evaluated the prevalence of multidrug resistant strains of* Salmonella* Infantis in broiler flocks in Slovenia and reported that* Salmonella* Infantis persistence on broiler farms seems to be more related to its widespread occurrence in the broiler production chain and to ineffective disinfection protocols than to its ability to form biofilm. L. Ketema* et al.* studied the prevalence and antimicrobial susceptibility profile of* Salmonella* serovars isolated from slaughtered cattle in Addis Ababa, Ethiopia, and detected multidrug resistant strains that pose a major public health concern, implying the need for a strict biosecurity and regulation of antimicrobial use.

M. Projahn* et al.* reviewed interventions against Enterobacteriaceae in broiler processing and concluded that none of the procedures was able to totally eradicate Enterobacteriaceae from the broiler carcasses, highlighting the need to develop intervention measures to prevent contamination with extended-spectrum beta-lactamase Enterobacteriaceae, thus avoiding the exposure of humans and the further release of antibiotic resistance into the environment.

Several studies have addressed the genetic diversity occurring in food pathogens. M. Bilung* et al.* studied the prevalence, genetic diversity (ERIC- and BOX-PCR), and antibiotic resistance profile of* Listeria* spp. and* Listeria monocytogenes* at the farm level and concluded that hygienic measures are needed to reduce the spread of* Listeria* along the food chain. A. Dekowska and coworkers characterised the genetic diversity of* Alicyclobacillus* strains and concluded that RFLP analysis of the 16S rRNA and* rpoB* genes, as well as* vdc* region, can be used for identification and intraspecies differentiation of* Alicyclobacillus*. A. Bashir* et al.* studied the ability of pet food factory, clinical and veterinary* Salmonella* isolates to form biofilms and reported that, although biofilm formation is an important mechanism of environmental persistence in the food manufacturing environments, there is no evidence of an enhanced biofilm-producing phenotype in factory persistent strains. A. T. S. Lopes and colleagues evaluated the advantages and disadvantages of using a multiplex real-time PCR to quantify* Salmonella* spp.,* E. coli*, and* Staphylococcus aureus* in different food matrices. E. Walecka-Zacharska and collaborators studied the effect of heat stress on the invasiveness ability of* L. monocytogenes* and reported that exposure to heat stress significantly decreased the invasiveness of* L. monocytogenes* strains.

Food safety is affected by food processing technologies, the use of preservatives, food packaging systems, and food transportation, among other factors that modulate the food microbiome. B. A. Zullo* et al.* demonstrated that olive oil polar phenols can prevent the survival of coliform bacteria in virgin olive oil. F. A. Obeng and coworkers evaluated the microbiological quality of tomatoes sold at central markets in Ghana and detected a high contamination levels in both spoilt and fresh tomatoes, which might have been caused by poor sanitation, improper handling, or transportation from the farms to the markets. J. Nasilowska and colleagues used high isostatic pressure technology to assure the microbial safety of long-term stored vegetable juices. A. Bah* et al.* evaluated the inhibitory effect of* Lactobacillus plantarum* and* Leuconostoc mesenteroides* strains against foodborne pathogens in artificially contaminated fermented tomato juices. The tested strains are potential starters for developing nutritious and safe fermented tomato juice products, because they showed high survival rates, while the numbers of pathogenic bacteria, yeasts, and moulds decreased drastically throughout storage.

Regarding the microbiology of food fermentations, O. J. Oumer and D. Abate performed comparative studies of pectinase production by* Bacillus subtilis* in submerged and solid-state fermentations using agroresidues and reported that the maximum pectinase production was attained using wheat bran. G. Fan* et al.* improved the production of ethyl acetate in Baijiu using mixed culture fermentations with* Wickerhamomyces anomalus* and* Saccharomyces cerevisiae*.

Probiotics have been a hot issue in the health industry for some years and several papers were submitted on this topic. Y. Nazir and colleagues reviewed the potential preventive and therapeutic role of probiotics for cancer, high serum cholesterol, and allergic and HIV diseases as well as providing their possible mechanisms of actions. D. Zielinska and D. Kolozyn-Krajewska revised the probiotic properties of food lactic acid bacteria. A. Peirotén* et al.* evaluated the technological properties of bifidobacterial strains shared by mother and child and considered two strains of* Bifidobacterium breve* and* Bifidobacterium bifidum* to be good candidates as adjunct cultures in cheeses as potential probiotics. O.-A Praepanitchai and coworkers studied the survival and behaviour of encapsulated* Lactobacillus plantarum* probiotics under different processing conditions in pasteurized mango juice and found out that most bacteria did not survive at temperatures above 50°C nor pH values below 3. Additionally, the survival of probiotic cells was higher with hybrid hydrogel beads containing alginate and soy protein.

N. Salameh and colleagues studied the* in vitro* antilipase and anti-alpha-amylase effect of the volatile oils or volatile organic compounds (VOCs) of* Micromeria fruticosa* ssp.* Serpyllifolia*, a plant that is consumed as an infusion in Palestine, and reported that their phytochemicals provide different potential biological activities. In effect, moulds produce alpha-amylases to utilise starch, and thus these volatile oils could be used as antifungals in starchy foods, for they leave no taste or odour.

Submitting authors come from 15 different countries, six European (Poland, Slovenia, Germany, Italy, UK, and Spain), and nine non-European (China, Ethiopia, Malaysia, South Korea, Brazil, Palestine, Ghana, Tunisia, and Thailand).

We are pleased to introduce this special issue, which includes 22 papers on very diverse topics within food microbiology and we wish that the readers find this issue of relevance and importance for their research.

